# A Review of the Current Trends and Future Perspectives of Robots in Colorectal Surgery: What Have We Got Ourselves Into?

**DOI:** 10.7759/cureus.77690

**Published:** 2025-01-20

**Authors:** Reda H Mithany, Amarah Shaikh, Sreedutt Murali, Ahmad Rafique, Peter S Bebawy, Prashant Girijavallabhan Nair, Wafaa Ramadan, Momen Abdelglil, Aayush Gupta, Md Abu Sayed, Mohamed Ismaiel

**Affiliations:** 1 Laparoscopic Colorectal Surgery, Kingston Hospital NHS Foundation Trust, Kingston Upon Thames, GBR; 2 General Surgery, Kingston Hospital NHS Foundation Trust, Kingston Upon Thames, GBR; 3 Colorectal Surgery, Addenbrooke's Hospital, Cambridge University Hospitals NHS Foundation Trust, Cambridge, GBR; 4 General and Colorectal Surgery, Torbay and South Devon NHS Foundation Trust, Torquay, GBR; 5 School of Medicine, Menya University, Menya, EGY; 6 General Surgery, Friarage Hospital, North Yorkshire, GBR; 7 Surgery, Hillingdon Hospital, Uxbridge, GBR; 8 Pediatric Surgery, Mansoura University Children Hospital, Mansoura, EGY; 9 Colorectal Surgery, Torbay Hospital, Torbay, GBR; 10 General Surgery, University Hospital Coventry and Warwickshire NHS Trust, Coventry, GBR; 11 Colorectal Surgery, The James Cook University Hospital, Middlesbrough, GBR

**Keywords:** advances, artificial intelligence (ai) in surgery, clinical outcomes, colorectal cancer, robotic surgery

## Abstract

Robotic colorectal surgery represents a significant advancement in the management of complex colorectal conditions, offering enhanced precision, safety, and improved patient outcomes. It is widely utilised for colorectal cancer, inflammatory bowel disease, diverticular disease, and rectal prolapse, with key benefits such as 3D visualisation, superior dexterity, and precise navigation in confined spaces. These advantages contribute to lower conversion rates to open surgery, faster recovery, reduced pain, and shorter hospital stays. This narrative review analysed recent peer-reviewed literature, focusing on technological advancements, clinical outcomes, and emerging challenges in robotic colorectal surgery. Findings highlight improved oncological precision, faster recovery, and fewer complications, driven by innovations like AI-guided decision-making and advanced robotic platforms. However, issues such as prolonged operative times, high costs, and steep learning curves remain. Future efforts should prioritise integrating AI, enhancing surgeon training, and addressing cost barriers to maximise the potential of robotic colorectal surgery in improving patient care.

## Introduction and background

Colorectal surgery has seen significant advancements in recent years, particularly with the emergence of robotic-assisted surgery as a transformative technology. This innovative approach addresses many limitations of traditional open surgeries and laparoscopic techniques, offering enhanced precision, safety, and effectiveness for colorectal procedures. Robotic surgery has gained widespread acceptance in hospitals and medical centres around the world, expanding its applications from cancer surgeries to a broader range of colorectal conditions. Recent literature highlights numerous benefits of robotic-assisted colorectal surgery, including improved surgical precision, shorter hospital stays and recovery times, and lower complication rates [1,2].

A study published in 2024 in the World Journal of Surgical Oncology demonstrated that patients undergoing robotic colectomies for colon cancer experienced shorter hospital stays and lower complication rates than those undergoing laparoscopic procedures [3]. The study also revealed that robotic surgery resulted in more lymph nodes harvested, leading to more accurate cancer staging.

An essential development in robotic colorectal surgery is the introduction of advanced robotic platforms. For example, unlike traditional laparoscopic surgery, which relies on rigid instruments and 2D visualisation, robotic systems offer 3D high-definition imaging and wristed instruments for enhanced manoeuvrability. This enables precise dissection and tissue handling in challenging areas such as the pelvis [4]. These capabilities are particularly valuable in complex colorectal procedures, where the margin for error is minimal.

Recent articles have also examined the long-term oncological outcomes of robotic colorectal surgery. A study published in Nature Scientific Reports compared the early surgical outcomes of robotic and laparoscopic approaches in colorectal cancer resections. The findings suggested that the robotic approach is safe and feasible, with comparable intra-operative and peri-operative morbidity and mortality rates to those associated with laparoscopic techniques [5].

Despite these advancements, challenges remain in the widespread adoption of robotic colorectal surgery. The high costs associated with robotic platforms pose a significant barrier, especially in low-resource settings. Additionally, the learning curve for surgeons requires extensive training to achieve proficiency with robotic systems [6].

Emerging technologies are expected to further enhance robotic colorectal surgery. The integration of artificial intelligence, as well as augmented and virtual reality, holds promise for improving preoperative planning, intraoperative navigation, and real-time decision support [7]. Robotic surgery is already a reality in many hospitals, emphasising the need for essential bioethical reflections on the relationship between healthcare professionals, robotic systems, and patients [8].

## Review

Methodology

This review systematically analyzed peer-reviewed literature on robotic colorectal surgery, focusing on studies published within the last four years (2020-2024) to highlight recent advancements and trends. Databases such as PubMed, Scopus, and Web of Science were searched using keywords like ("robotic colorectal surgery" OR "robotic-assisted colorectal surgery") AND ("outcomes" OR "cost" OR "AI" OR "learning curve") AND ("randomized controlled trial" OR "cohort study").

Studies were included if they were peer-reviewed, published in the last four years, and focused on robotic colorectal surgery, technological advancements, clinical outcomes, or related challenges. Articles unrelated to colorectal surgery or lacking original data were excluded.

Relevant abstracts and full texts were screened to ensure alignment with the review's objectives. Key data on clinical benefits, emerging technologies, ethical considerations, and future trends were extracted and analyzed to provide an up-to-date perspective on robotic colorectal surgery.

History of robotic colorectal surgery

The journey of robot-assisted medical procedures started in 1985 using the PUMA (Programmable Universal Manipulation Arm) 560 robotic arm (Unimation, Danbury, CT, US) for a CT-guided stereotactic brain biopsy [9]. The stage for broader surgical applications was set after the approval of AESOP (Automated Endoscopic System for Optimal Positioning; Computer Motion, Santa Barbara, CA, USA), a robotic camera holder, by the FDA (Food and Drug Administration) in 1994 [10]. The first robotic colorectal surgery was performed in 2001 by Cadière and Himpens, who pioneered the use of robotic technology for transanal rectal resection. Shortly after, Weber et al. (2002), using the da Vinci Surgical System (Intuitive Surgical, Inc., Sunnyvale, CA, US), reported the first robotic right and sigmoid colectomies for benign disease [11].

In 2003, Delaney et al. published a comparison of robotic-assisted laparoscopic colectomy to standard laparoscopic approaches [12]. The first robotic-assisted rectopexy for rectal prolapse and robot-assisted anterior resections for rectal cancer were documented in the same year [13]. D'Annibale et al. documented 53 cases of robotic colorectal surgeries, including those for malignant disease, in 2004, which reflects the extensive progression of robotic surgery in colorectal surgery [14].

Pigazzi et al. introduced robotic total mesorectal excision in 2006, marking a pivotal advancement in rectal cancer management [15]. In the same year, robotic abdominoperineal resection was also performed in Asia [16,17].

In 2008, a study by Pinoglio et al. showed a lower complication rate of robotic colorectal surgeries than laparoscopic procedures [18]. The following years saw rapid adoption of robotic surgery, culminating in the establishment of dedicated robotic colorectal programs such as the one at East Lancashire Hospitals NHS Trust in 2017 [19].

**Figure 1:**
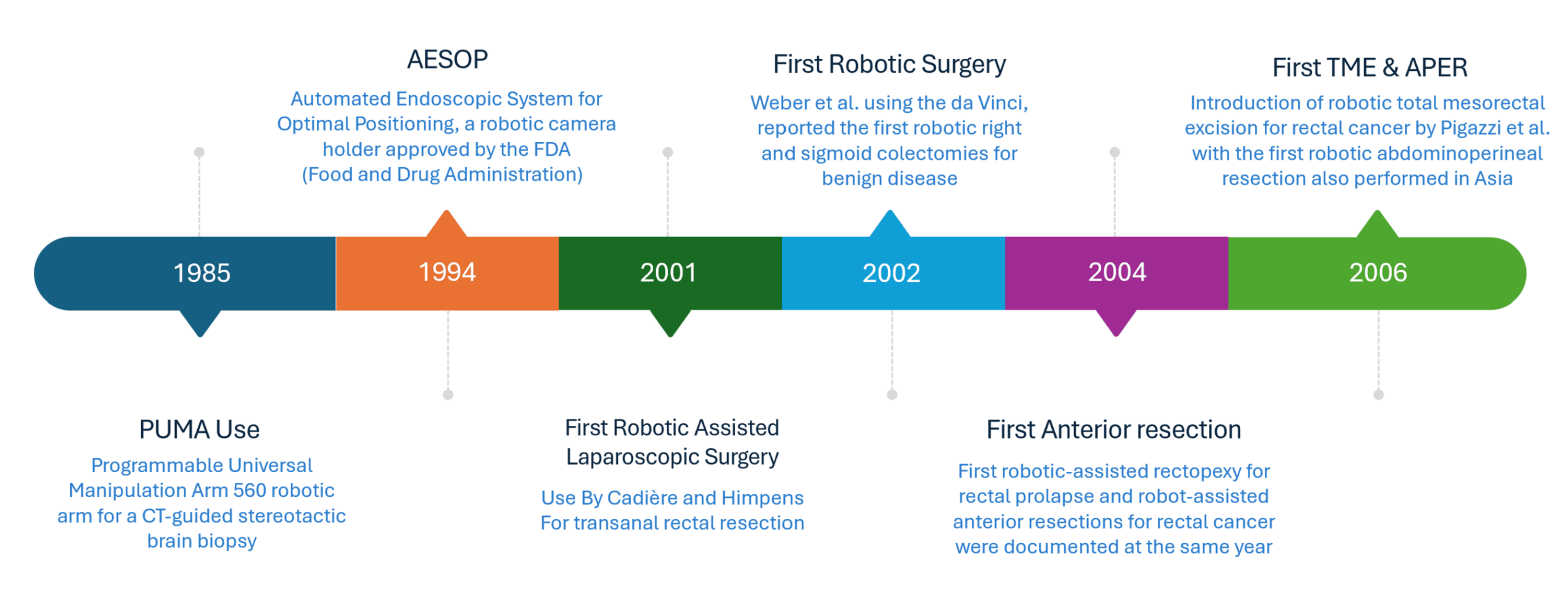
An illustrated timeline of key milestones in the evolution of robotic-assisted surgery, highlighting pivotal advancements and their impact on clinical practice Figure Credits: Reda Harby Mithany Source: [9-20]

The da Vinci 5 Surgical System was introduced in 2024, incorporating advanced features such as enhanced ergonomics, better instrumentation, and integrated artificial intelligence tools. These advancements reflect the continuous evolution of robotic colorectal surgery, addressing limitations while providing safer and more effective surgical outcomes (Figure 1) [20].

Technological innovations in robotic colorectal surgery

Robotic colorectal surgery has achieved significant technological advancements, resulting in more precise, efficient, and safer procedures over the last four years. The landscape of colorectal surgery has been reshaped with innovations in robotic platforms, the integration of artificial intelligence (AI), advancements in instrumentation, and improvements in training methods. This evolution has led to better clinical outcomes, particularly oncological and postoperative recovery, while also addressing the robotic surgery learning curve [21].

Robotic Platforms and Systems

The Da Vinci Xi and SP (Single Port) systems have seen considerable enhancements, making them more effective in performing complex colorectal surgeries. The Da Vinci Xi improves oncological outcomes by reducing positive resection margins in total mesorectal excision (TME) for rectal cancer using its 3D high-definition optics and wristed instruments, allowing for enhanced precision in delicate procedures [22]. With its single-port approach, the Da Vinci SP system enables minimally invasive surgery through a small incision, reducing postoperative pain and enhancing recovery times [23].

In recent years, new robotic platforms like Versius (CMR Surgical, Cambridge, UK) and Senhance (Asensus Surgical Inc., Durham, NC, US) have emerged, offering significant advantages over traditional systems. Versius features a modular design that provides greater flexibility and enhanced comfort for surgeons. Meanwhile, the Senhance system improves surgical precision by incorporating haptic feedback and 3D vision. Studies indicate that the outcomes of these newer systems are comparable to those of the Da Vinci system, suggesting that competition in the field could drive further innovation and potentially reduce costs [24].

AI and Machine Learning Integration

Integrating AI and machine learning into robotic colorectal surgery has opened new avenues for enhancing surgical precision and improving patient outcomes. Large datasets are utilised to train machine learning models, particularly through supervised learning, where the algorithms learn to identify patterns and make predictions based on historical surgical data. These models are designed to label data, using information from previous surgeries, such as patient demographics, tumor locations, and surgical techniques, to teach the algorithm. Over time, as they encounter more data, these models are refined, leading to greater accuracy in predicting surgical outcomes [25].

Continuously adapting and improving predictions is a crucial aspect of machine learning. The model evolves and refines its predictions as new data is collected from each surgery. This is especially evident in AI-assisted navigation, where the system, initially based on basic patterns, provides guidance that becomes more accurate over time as it processes a wider range of patient data and surgical scenarios. Additionally, AI predicts postoperative complications and offers real-time decision support during surgery. Machine learning algorithms assess the likelihood of adverse events, such as anastomotic leaks, infections, or bleeding, by analyzing patient-specific data, including medical history, vital signs, and intra-operative factors. Over time, these models enhance their predictive accuracy, enabling more personalised and proactive care by learning from the outcomes of previous patients. The ability to predict complications with greater precision is vital for improving patient safety and reducing the overall risk of surgical failure, as AI systems continue to advance with each surgical case [26].

AI models are increasingly being developed to predict long-term outcomes, such as recurrence rates and survival probabilities, by analyzing extensive patient data collected over long periods. These predictions assist surgeons in creating more personalised postoperative care plans and follow-up strategies, resulting in individualised and more effective treatment for each patient [27].

Robotic Instrumentation and Advanced Imaging

The success of robotic colorectal surgery is largely due to advancements in instrumentation and imaging technology. Improved 3D optics enhance the visualisation of the surgical site, allowing for better orientation, particularly in tight spaces like the pelvis. When paired with wristed instruments, these advanced optics give surgeons exceptional dexterity, reducing the risk of tremors and increasing precision during delicate procedures [28]. Additionally, research has shown that robotic surgery, due to its advanced optics and instrumentation, leads to better nerve preservation during rectal surgeries. This preservation directly influences postoperative outcomes, such as sexual and urinary function, which are crucial for the patient’s quality of life [29].

Furthermore, the integration of intraoperative navigation and imaging technologies, such as real-time CT, MRI, and ultrasound, has transformed robotic colorectal surgery. These tools enable surgeons to visualise tumours and surrounding tissues in real time, improving the accuracy of resections and reducing the risk of positive margins [30].

Clinical outcomes

Robotic colorectal surgery has been shown to improve clinical outcomes. Studies consistently demonstrate that complete mesorectal excision (CME) performed with robotic assistance leads to better adherence to oncological principles such as maintaining clear resection margins and reducing the number of positive lymph nodes postoperatively [31]. Numerous studies have shown that robotic colorectal surgery results in significantly fewer complications, such as anastomotic leaks, as compared to laparoscopic surgery, thereby improving overall patient outcomes [32]. Furthermore, the robotic approach shortens hospital stays and enhances recovery time [33].

Another study has concluded that robotic surgery for rectal cancer does not provide significant oncologic or clinical benefits compared to laparoscopic surgery, despite its higher cost. The study evaluated long-term outcomes in 217 patients with stage I-III rectal cancer over a median follow-up of 58 months. It found no important differences in 5-year overall survival, disease-free survival, or local recurrence rates between robotic and laparoscopic surgeries. Robotic surgery, however, was associated with a higher conversion rate, shorter hospital stays, and costs approximately 2.34 times greater than laparoscopic surgery [34].

Training and mentorship****

One of the most successful models in robotic surgery training is the "Train-the-Trainer" program, which emphasises the development of expert trainers who can share their knowledge and experience with less experienced surgeons. This approach has been widely implemented in robotic surgery education to ensure that experienced surgeons possess not only technical expertise but also effective teaching skills. The Train-the-Trainer model in robotic colorectal surgery has significantly improved the proficiency of both trainers and trainees by refining teaching methods and providing constructive feedback. Trainers use structured evaluation tools to assess trainees' performance and adapt their teaching strategies accordingly, ensuring that new surgeons develop competence in both technical skills and decision-making [35].

Another highly effective program is the Fundamental Skills in Robotic Surgery (FSRS) course, which has been successful in equipping surgeons with foundational robotic skills. This program combines theoretical learning, simulated practice, and hands-on training in robotic surgery [36].

**Figure 2:**
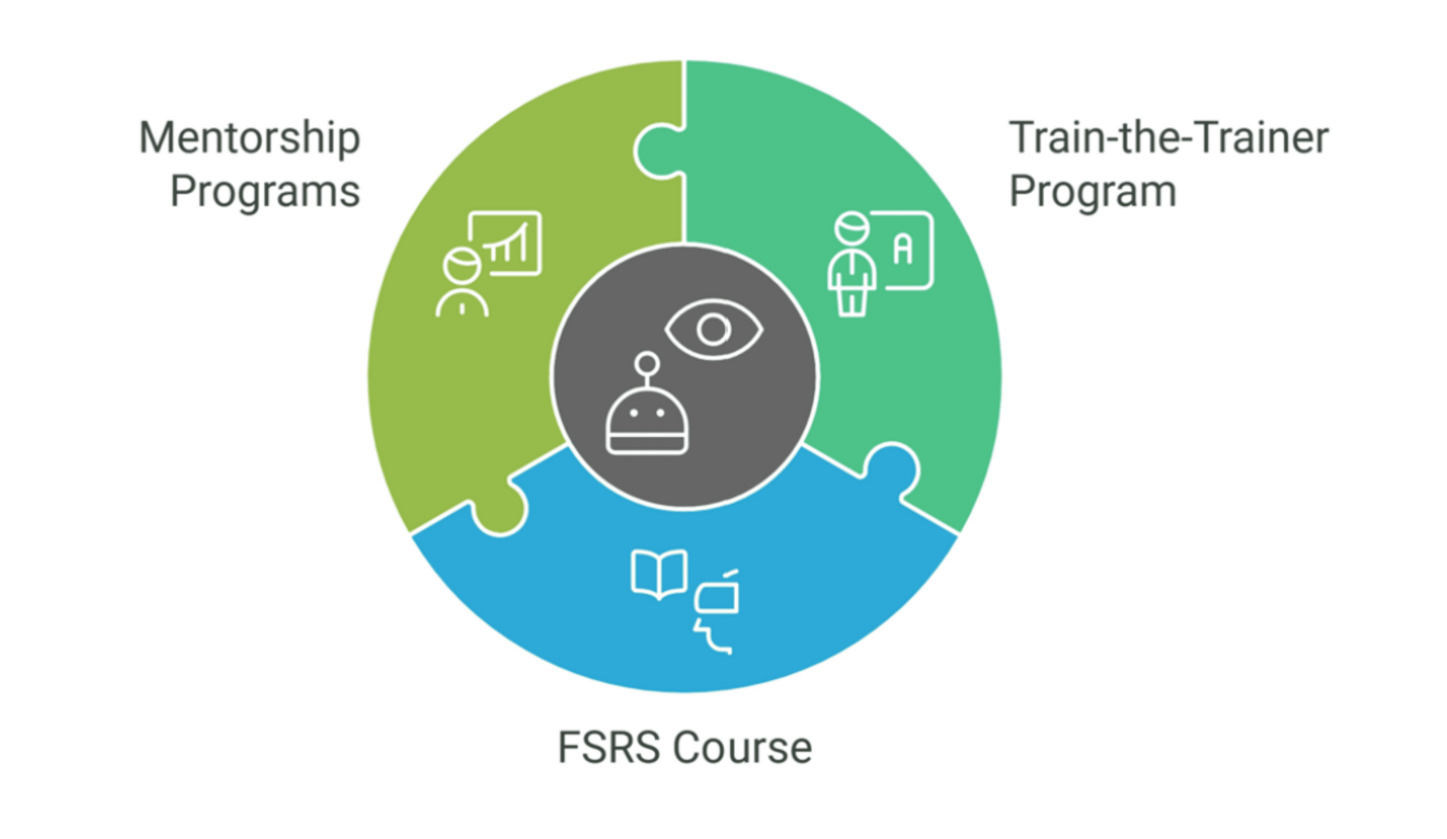
Robotic surgery training programs FSRS: Fundamental Skills in Robotic Surgery Figure Credits: Momen Abdelglil Source: [35-37]

Mentorship programs also play a crucial role in surgical training, particularly in fields like colorectal surgery, where experience is essential. The mentor-mentee model, in which a seasoned surgeon guides a less experienced one, is widely used in surgical training. Evidence indicates that mentorship enhances not only technical skills but also decision-making abilities (Figure 2) [37]. 

Cost-effectiveness analysis in robotic colorectal surgery

Comparative Costs Between Robotic and Laparoscopic Surgery

Studies comparing the costs of robotic colorectal surgery to laparoscopic surgery have highlighted several factors that contribute to the overall financial burden. Robotic surgery has substantial upfront costs, as the robotic system can range from $1.5 million to $2 million for the device itself. Additionally, there are maintenance and training costs, which can pose challenges for many institutions [38].

Another study found that robotic colorectal surgery is associated with fewer instances of anastomotic leaks and other complications, such as wound infections, as compared to laparoscopic procedures. These reductions in complications lead to lower readmission rates, fewer surgical revisions, and a reduced need for long-term follow-up for various operations. Consequently, despite the higher initial costs, robotic surgery often results in a lower total cost per patient when factoring in complications and readmissions [33].

*Impact of Reduced Hospital Stay and Complications on Cost*****

One of the most significant economic benefits of robotic colorectal surgery lies in the reduction of postoperative complications and hospital stay duration. Robotic surgery, due to its minimally invasive nature and superior precision, often leads to faster recovery times. This means that patients typically experience less postoperative pain and can return to normal activities sooner, reducing the need for prolonged hospital care [39].

**Figure 3:**
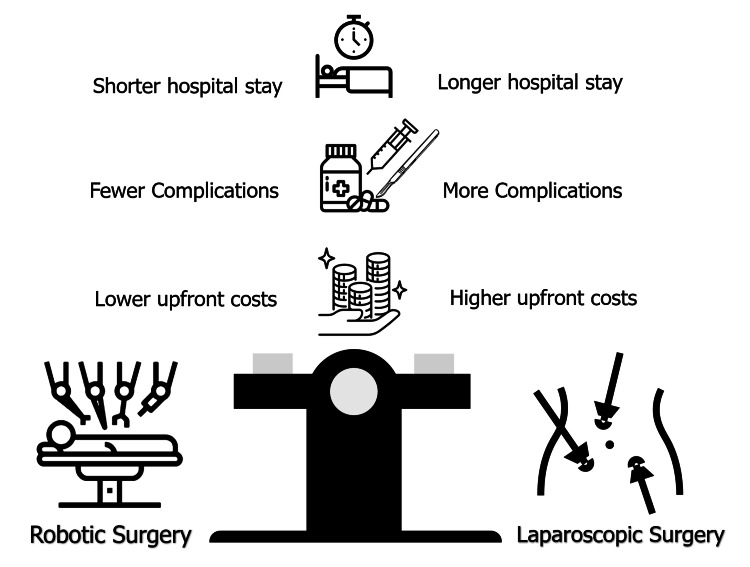
Evaluating cost-effectiveness between robotic and laparoscopic surgery Figure Credits: Reda Harby Mithany Source: [39,40]

Robotic colorectal surgery was found to be associated with a 35% reduction in complication rates, including wound infections and anastomotic leaks, compared to laparoscopic surgery. With fewer complications, hospitals can avoid additional expenses related to re-admission, extended care, and postoperative interventions at different levels. Additionally, reduced recovery time and fewer complications result in lower overall healthcare costs, making robotic surgery an economically viable option despite its higher initial investment (Figure 3) [40].

The economic impact of robotic surgery is a topic of considerable debate. While robotic surgery involves higher initial costs due to the expense of equipment, it can ultimately be cost-effective due to reduced complication rates, shorter hospital stays, and faster recovery [41].

Emerging Low-Cost Robotic Platforms and Their Potential

Several companies are working to develop affordable robotic platforms that can reduce the financial barriers for hospitals in low-income regions, potentially facilitating the spread of robotic surgery. One example is the RAS-0 system, created by MIRA Robotics (Saitama, Japan), which serves as a low-cost alternative to more expensive systems like the Da Vinci. It provides essential features, such as 3D visualisation and wristed instruments, at a fraction of the cost of top-tier systems, making it an appealing option for resource-limited hospitals [42,43].

Additionally, mobile robotic units are being explored as a viable solution for delivering high-quality care in underserved areas. These portable systems can be transported between hospitals and colorectal centres, minimising the need for significant investments in each facility. This flexibility in deployment could greatly increase access to robotic surgery in countries with limited resources and in low-income regions [44].

Future of robotic surgery

Personalised and Precision Surgery

Personalised and precision surgery is becoming a significant trend in the field of robotic colorectal surgery. This approach involves tailoring surgical interventions based on the unique anatomical features of each patient, which can be evaluated preoperatively using advanced imaging techniques such as CT scans, MRIs, and ultrasound. By integrating artificial intelligence, robotic systems can analyze these images and assist surgeons in developing a patient-specific surgical plan. This enhances the precision of the surgery and reduces the likelihood of complications [10,45].

Miniaturisation and Flexible Robotics

The trend of miniaturisation in robotic systems represents a promising development in the field of surgery. Smaller and more flexible robotic instruments could greatly improve a surgeon's ability to perform complex procedures in confined spaces and various situations. New robotic systems are being designed to be more compact and adaptable, which enhances mobility and precision during colorectal surgeries. For instance, flexible robotic tools, such as those developed by companies like Medrobotics (Raynham, MA, US) offer flexibility similar to traditional laparoscopic instruments while providing the added advantage of robotic precision. This innovation helps overcome some of the limitations associated with current surgical systems [46,47].

## Conclusions

Robotic colorectal surgery has made significant advancements in recent years, particularly with innovations in platforms like the Da Vinci Xi and SP systems. Emerging technologies, such as AI integration and enhanced imaging, are also contributing to these improvements. As a result, surgical precision has increased, recovery times have decreased, and complications have been minimised, establishing robotic surgery as a superior choice for safety and efficiency.

Looking ahead, future advancements may include miniaturised systems, AI-driven personalised surgeries, and affordable robotic platforms, all aimed at making these technologies more accessible and effective. Despite challenges such as high costs and the need for specialised training, robotic colorectal surgery continues to evolve, promising transformative improvements in patient care, disease outcomes, and global adoption. To ensure its widespread implementation, future efforts must focus on reducing costs, improving accessibility, and fostering collaboration between surgeons, engineers, and policymakers.
